# A cross-sectional study of ophthalmologic examination findings in 5385 Koreans presenting with intermittent exotropia

**DOI:** 10.1038/s41598-023-28015-2

**Published:** 2023-01-24

**Authors:** Dae Hee Kim, Jae Ho Jung, Mi Young Choi, Jeong-Min Hwang, Su Jin Kim, Yeon-hee Lee, Sueng-Han Han, Dong Gyu Choi, The Korean Association of Pediatric Ophthalmology and Strabismus (KAPOS), Seung-Hee Baek, Hee-young Choi, Dong Gyu Choi, Dae Hee Kim, Dong Cheol Lee, Se-Youp Lee, Han Woong Lim, Hyun Taek Lim, Key Hwan Lim, Won Yeol Ryu, Hee Kyung Yang, Hee-young Choi, Hyun Taek Lim, Jae Ho Jung, Seung-Hee Baek, Mi Young Choi, Jeong-Min Hwang, Su Jin Kim, Yeon-hee Lee, Sueng-Han Han, Shin Hae Park, Haeng-Jin Lee, Sook-Young Kim, Se-Youp Lee, Hyo Jung Gye, So Young Kim, Sun Young Shin, Jihyun Park, Won Yeol Ryu, Hye Sung Park, Dae Hee Kim, Hae Jung Paik, Dong Gyu Choi, Joo Yeon Lee, Hee Kyung Yang, Shin Yeop Oh, Soo Jung Lee, Seung Ah Chung, Jin Choi, Sei Yeul Oh, Mirae Kim, Young-Woo Suh, Nam Yeo Kang, Hae Ri Yum, Sun A. Kim, Hyuna Kim, Jinu Han, Yoonae A. Cho, Hyunkyung Kim, Helen Lew, Dong Cheol Lee, Sang Hoon Rah, Yung-Ju Yoo, Key Hwan Lim, Hyosook Ahn, Ungsoo S. Kim, Jung Ho Lee, Hokyung Choung, Seong-Joon Kim, Hyeshin Jeon, Hyun Jin Shin, So Young Han, Hwan Heo, Soochul Park, Songhee Park, Sung Eun Kyung, Changzoo Kim, Kyung-Ah Park, Eun Hye Jung, Eun Hee Hong, Han Woong Lim, Daye Choi, Youn Joo Choi, Nam Ju Moon, In Jeong Lyu, Soon Young Cho

**Affiliations:** 1 Department of Ophthalmology, Kim’s Eye Hospital, Seoul, Korea; 2 Department of Ophthalmology, Seoul National University College of Medicine, Seoul National University Hospital, Seoul, Korea; 3 Department of Ophthalmology, Chungbuk National University College of Medicine, Chungbuk National University Hospital, Cheongju, Korea; 4 Department of Ophthalmology, Seoul National University College of Medicine, Seoul National University Bundang Hospital, Seongnam, Korea; 5 Department of Ophthalmology, Pusan National University School of Medicine, Yangsan Pusan National University Hospital, Yangsan, Korea; 6 Department of Ophthalmology, Chungnam National University College of Medicine, Chungnam National University Hospital, Daejeon, Korea; 7 Department of Ophthalmology, Yonsei University College of Medicine, Severance Eye Hospital, Seoul, Korea; 8 Department of Ophthalmology, Hallym University College of Medicine, Kangnam Sacred Heart Hospital, 1, Singil-Ro, Yeongdeungpo-Gu, Seoul, 07441 Republic of Korea; 9 Department of Ophthalmology, Pusan National University Hospital, Busan, Korea; 10 Department of Ophthalmology, Asan Medical Center, Seoul, Korea; 11 Department of Ophthalmology, Seoul St. Mary’s Hospital, Seoul, Korea; 12 Department of Ophthalmology, Jeonbuk National University Hospital, Jeonju, Korea; 13 Department of Ophthalmology, Daegu Catholic University Medical Center, Daegu, Korea; 14 Department of Ophthalmology, Keimyung University DongSan Hospital, Daegu, Korea; 15 Department of Ophthalmology, Nune Eye Hospital, Seoul, Korea; 16 Department of Ophthalmology, Soonchunhyang University Cheonan Hospital, Cheonan, Korea; 17 Department of Ophthalmology, Nune Eye Hospital, Daegu, Korea; 18 Department of Ophthalmology, Dong-A University Hospital, Busan, Korea; 19 Department of Ophthalmology, Siloam Eye Hospital, Seoul, Korea; 20 Department of Ophthalmology, Gachon University Gil Medical Center, Incheon, Korea; 21 Department of Ophthalmology, Hallym University Sacred Heart Hospital, Anyang, Korea; 22 Department of Ophthalmology, Samsung Changwon Hospital, Changwon, Korea; 23 Department of Ophthalmology, Inje University Haeundae Paik Hospital, Busan, Korea; 24 Department of Ophthalmology, Ajou University Hospital, Suwon, Korea; 25 Department of Ophthalmology, Inje University Sanggye Paik Hospital, Seoul, Korea; 26 Department of Ophthalmology, Samsung Medical Center, Seoul, Korea; 27 Department of Ophthalmology, Korea University Ansan Hospital, Ansan, Korea; 28 Department of Ophthalmology, Bucheon St. Mary’s Hospital, Bucheon, Korea; 29 Department of Ophthalmology, Eunpyeong St. Mary’s Hospital, Seoul, Korea; 30 Department of Ophthalmology, Sungmo Eye Hospital, Busan, Korea; 31 Department of Ophthalmology, Soonchunhyang University Seoul Hospital, Seoul, Korea; 32 Department of Ophthalmology, Gangnam Severance Hospital, Seoul, Korea; 33 Department of Ophthalmology, Hangil Eye Hospital, Incheon, Korea; 34 Department of Ophthalmology, Bundang CHA Medical Center, Seongnam, Korea; 35 Department of Ophthalmology, Wonju Severance Christian Hospital, Wonju, Korea; 36 Department of Ophthalmology, Kangwon National University Hospital, Chuncheon, Korea; 37 Department of Ophthalmology, Ewha Womans University Mokdong Hospital, Seoul, Korea; 38 Daegu Premier Eye Center, Daegu, Korea; 39 Department of Ophthalmology, SMG-SNU Boramae Medical Center, Seoul, Korea; 40 Department of Ophthalmology, Konkuk University Medical Center, Seoul, Korea; 41 Department of Ophthalmology, Kangbuk Samsung Hospital, Seoul, Korea; 42 Department of Ophthalmology, Chonnam National University Hospital, Gwangju, Korea; 43 Department of Ophthalmology, Saevit Eye Hospital, Goyang, Korea; 44 Magok Dream Light Eye Clinic, Seoul, Korea; 45 Department of Ophthalmology, Kosin University Gospel Hospital, Busan, Korea; 46 Department of Ophthalmology, Nowon Eulji Medical Center, Seoul, Korea; 47 Department of Ophthalmology, Hanyang University Guri Hospital, Guri, Korea; 48 Department of Ophthalmology, Hanyang University Hospital, Seoul, Korea; 49 Department of Ophthalmology, Hallym University Kangdong Sacred Heart Hospital, Seoul, Korea; 50 Department of Ophthalmology, Chungang University Hospital, Seoul, Korea; 51 Department of Ophthalmology, Korea Cancer Center, Goyang, Korea; 52 Department of Ophthalmology, Dongguk University Hospital, Gyeongju, Korea

**Keywords:** Eye abnormalities, Ocular motility disorders

## Abstract

The Korean Intermittent Exotropia Multicenter Study (KIEMS) was a retrospective, cross-sectional and multicenter study for the investigation of intermittent exotropia involved 65 strabismus specialists from 53 institutions in Korea. Purpose of this study was to present ophthalmologic findings of intermittent exotropia from the KIEMS. Consecutive patients with intermittent exotropia of ≥ 8 prism diopters (PD) at distance or near fixation were included. Best-corrected visual acuity, cycloplegic refraction data, angles of deviation at several cardinal positions, ocular dominance, fusion control, oblique muscle function, and binocular sensory outcomes were collected. A total of 5385 participants (2793 females; age 8.2 years) were included. Non-dominant eye was more myopic than the dominant eye (− 0.60 vs. − 0.47 diopters, *P* < 0.001). Mean exodeviation angles were 23.5 PD at distance and 25.0 PD at near fixation. Basic type (86.2%) was the most, followed by convergence insufficiency (9.4%) and divergence excess (4.4%) types. Alternating ocular dominance and good fusion control were more common at near than at distance fixation. Good stereopsis at 40 cm was observed in 49.3% in Titmus stereo test (≤ 60 arcsec) and in 71.0% in Randot stereo test (≤ 63 arcsec). Intermittent exotropia was mostly diagnosed in childhood and patients with the condition showed relatively good binocular functions. This study may provide objective findings of intermittent exotropia in a most reliable way, given that the study included a large study population and investigated comprehensive ophthalmology examinations.

## Introduction

Intermittent exotropia is an outward drifting of either eye in a latent or intermittent form^1,2^. It is a predominant form of strabismus in East Asian countries^3–6^, including Korea^7,8^, and is also common in the United States^9^ and some European countries^10^. Although many clinical studies have been conducted on this common disease entity, many questions remain unanswered^11^. The interpretations of the results of many clinical studies on intermittent exotropia have been confusing owing to variable study settings, different study protocols, and the clinical variability of this condition^12^. Mostly, previous studies focused on the surgical results and included patients with relatively large angle of exotropia requiring surgery^13–17^, which might exclude the clinical findings of relatively small angle intermittent exotropia. Also, those studies reported various types of stereoacuity, binocularity, ocular dominance and fusion control tests^11,12^, which were not interchangeable for comparison. Most ophthalmologic examinations for diagnosis of intermittent exotropia depended largely on the examiners’ skill because those examinations can be performed only manually. To obtain comprehensive and convincing information about the clinical characteristics of intermittent exotropia, a large-scale study, regardless of clinical considerations, such as age, amount of exotropia angle, and necessity of surgical intervention, is needed. Also, the ophthalmologic examinations need to be conducted by strabismus specialists using a standardized protocol.

The Korean Intermittent Exotropia Multicenter Study (KIEMS) is a large-scale nationwide and multicenter study investigating the clinical features of intermittent exotropia using a standardized protocol. It was initiated by the Korean Association of Pediatric Ophthalmology and Strabismus (KAPOS), whose members are strabismus specialists. The KIEMS is one of the largest clinical studies on intermittent exotropia to date and is expected to present the overall features, including the subjective and objective features, of intermittent exotropia. This study was conducted to present the objective ophthalmologic findings from the KIEMS.

## Results

### Baseline characteristics of participants

A total of 5385 participants were included in this study with age of 8.2 ± 7.6 years (mean ± standard deviation; range, 0.3–106.7 years). The age distribution of all participants has been previously described^18^. The mean spherical equivalent (SE) was – 0.57 ± 1.89 diopters (D) (range, + 7.0 to − 12.88 D) in the right eye and -0.61 ± 1.96 D (range, + 8.75 to − 14.00 D) in the left eye (*P* = 0.666, paired t-test). The non-dominant eye at distance fixation tended to be more myopic than the dominant eye (SE: − 0.60 ± 1.98 vs. − 0.47 ± 1.74 D, *P* < 0.001, paired t-test) (Table 1).

**Table 1:** Baseline characteristics of participants.

	*n*	Mean	SD	Min	Max
Age, years	5385	8.2	7.6	0.3	106.7
Sex (female:male)	5385	2793:2592 (51.9%:48.1%)			
Spherical equivalent, diopters					
Right eye	4743	− 0.57	1.89	− 12.88	+ 7.00
Left eye	4248	− 0.61	1.96	− 14.00	+ 8.75
Best-corrected visual acuity (LogMAR)					
Right eye	4648	0.06	0.13	− 0.30	1.52
Left eye	4639	0.06	0.13	− 0.18	1.22
Exodeviation, prism diopters					
Distance	5354	23.5	8.8	0	85
Near	5358	25.0	9.3	0	90
Associated strabismus					
Vertical strabismus	5385	266 (4.9%)			
Dissociated vertical deviation	5385	35 (0.6%)			
Oblique dysfunction	4071	1136 (21.1%)			
Sensory status					
Good stereopsis	4340	2354 (54.2%)			
Fusion on the Worth four-dot test	3881	1924 (49.6%)			

Good stereopsis was defined as ≤ 60 arcsec in the Titmus test or ≤ 63 arcsec in the Randot test.

*SD* standard deviation, *Min* minimum, *Max* maximum.

Of the 5385 participants, 2793 (51.9%) were females and 2592 (48.1%) were males, showing a slight female predominance. Male participants were older than female participants (8.6 ± 7.3 vs. 7.8 ± 7.8 years, independent t-test, *P* < 0.001). According to the mean SE, the right and left eyes of male participants were more myopic than those of female participants (independent t-test, *P* < 0.001). Hyperopia (mean SE >  + 1 D), emmetropia (≤ + 1 and ≥ − 1 D), and myopia (< − 1 D) were observed in 12.0% (507/4219), 55.4% (2338/4219), and 32.6% (1374/4219) participants, respectively. The mean exodeviation angle in the primary position at distance fixation was 23.2 ± 9.0 PD in males, which was smaller than that in females (23.7 ± 8.6 PD) (independent t-test, *P* = 0.036). With respect to the mean exodeviation angle at near fixation, no sex difference was observed (25.0 ± 9.4 vs. 25.1 ± 9.3 PD, independent t-test, *P* = 0.543).

### Angles of exodeviation

The mean angle of exodeviation in the primary position was 23.5 ± 8.8 (range, 0–85) PD at distance fixation and 25.0 ± 9.3 (range, 0–90) PD at near fixation. Basic-type exotropia (difference between distant and near angles ≤ 10 PD), convergence insufficiency-type exotropia (near–distant angle < 10 PD), and divergence excess-type exotropia (distant–near angle > 10 PD) were observed in 86.2% (4599/5331), 9.4% (500/5331), and 4.4% (232/5331), respectively. Participants with convergence insufficiency-type exotropia were older than those with basic- and divergence excess-type exotropia (independent t-test, Bonferroni corrected *P* < 0.001). The exodeviation angles in the secondary and head-tilted positions were smaller than those in the primary position (Table 2, paired t-test, *P* < 0.001).

**Table 2:** Exodeviation angles according to gaze or head positions.

Gaze (or head) positions	*n*	Mean	SD	Min	Max	*P* value^a^
Distance						
Primary	5354	23.5	8.8	0	85	
Secondary						
Up gaze	3894	23.3	8.9	0	85	< 0.01
Down gaze	3889	22.5	8.8	0	85	< 0.01
Right gaze	3890	22.2	8.8	0	85	< 0.01
Left gaze	3892	22.1	8.9	0	85	< 0.01
Head tilted						
Right	2506	21.1	10.4	0	106	< 0.01
Left	2533	20.8	10.4	0	85	< 0.01
Near						
Primary	5358	25.0	9.3	0	90	< 0.01

Values are in prism diopters.

*SD* standard deviation, *Min* minimum, *Max* maximum.

^a^Compared with the distant exotropia angle in the primary position (paired t-test).

Lateral incomitance was present in 2.3% (95/4164) in right gaze and in 2.0% (83/4166) in left gaze. Lateral incomitance in both right gaze and left gaze was present in 1.5% (63/4163). The A and V patterns of exotropia were observed in 0.9% (35/3889) and 1.1% (44/3889), respectively.

### Ocular dominance and fusion control

Ocular dominance in the right or left eye was present in 51.7% (29.1% for the right eye, 22.6% for the left eye, 2407/4655) at distance fixation and in 39.0% (22.1% for the right eye, 16.9% for the left eye, 1725/4422) at near fixation. Alternating ocular dominance was observed in 48.3% (2248/4655) and 61.0% (2697/4422) at distance and near fixation, respectively. The ratio of alternating ocular dominance was significantly higher at near than at distance fixation (*P* < 0.001, Pearson’s chi-square test).

In the assessment of fusion control, the proportion of participants with good and fair control was 27.6% (1336/4835) and 41.6% (2010/4835) at distance fixation and 42.6% (1977/4641) and 37.1% (1723/4641) at near fixation, respectively. The proportion of participants who showed poor fusion control was 30.8% (1489/4835) at distance fixation and 20.3% (941/4641) at near fixation. Fusion control was better at near than at distance fixation (*P* < 0.001, Pearson’s chi-square test).

### Associated strabismus

Inferior oblique overaction (IOOA) was present in 25.5% of the participants (1092/4278), whereas superior oblique overaction (SOOA) was observed in only 6.0% (249/4120). Bilateral IOOA and SOOA (15.5% and 3.4%, respectively) were more common than unilateral IOOA and SOOA (10.0% and 2.6%, respectively). In contrast, inferior and superior oblique under actions were relatively rare (0.4% and 2.5%, respectively) (Table 3). Vertical deviation of ≥ 5 PD in the primary position was present in 4.9% (266/5385) (Table 1). Dissociated vertical deviation was present in 0.6% of the participants (35/5385) (Table 1).

**Table 3:** Inferior and superior oblique muscle underaction/overaction.

	*n*	Underaction	Overaction
Unilateral IO	4278	19 (0.4%)	429 (10.0%)
Bilateral IO		2 (0.0%)	663 (15.5%)
Unilateral SO	4120	89 (2.2%)	109 (2.6%)
Bilateral SO		14 (0.3%)	140 (3.4%)

*IO* inferior oblique, *SO* superior oblique.

### Sensory status evaluations

In the Worth four-dot test at 6 m, 49.6% of the participants (1924/3881) saw four lights, which was interpreted as “fusion” if normal retinal correspondence existed; 35.7% (1385/3881) saw two or three lights, recorded as “suppression”; and 14.7% (572/3881) saw five lights, recorded as “diplopia.”

**Figure 1:**
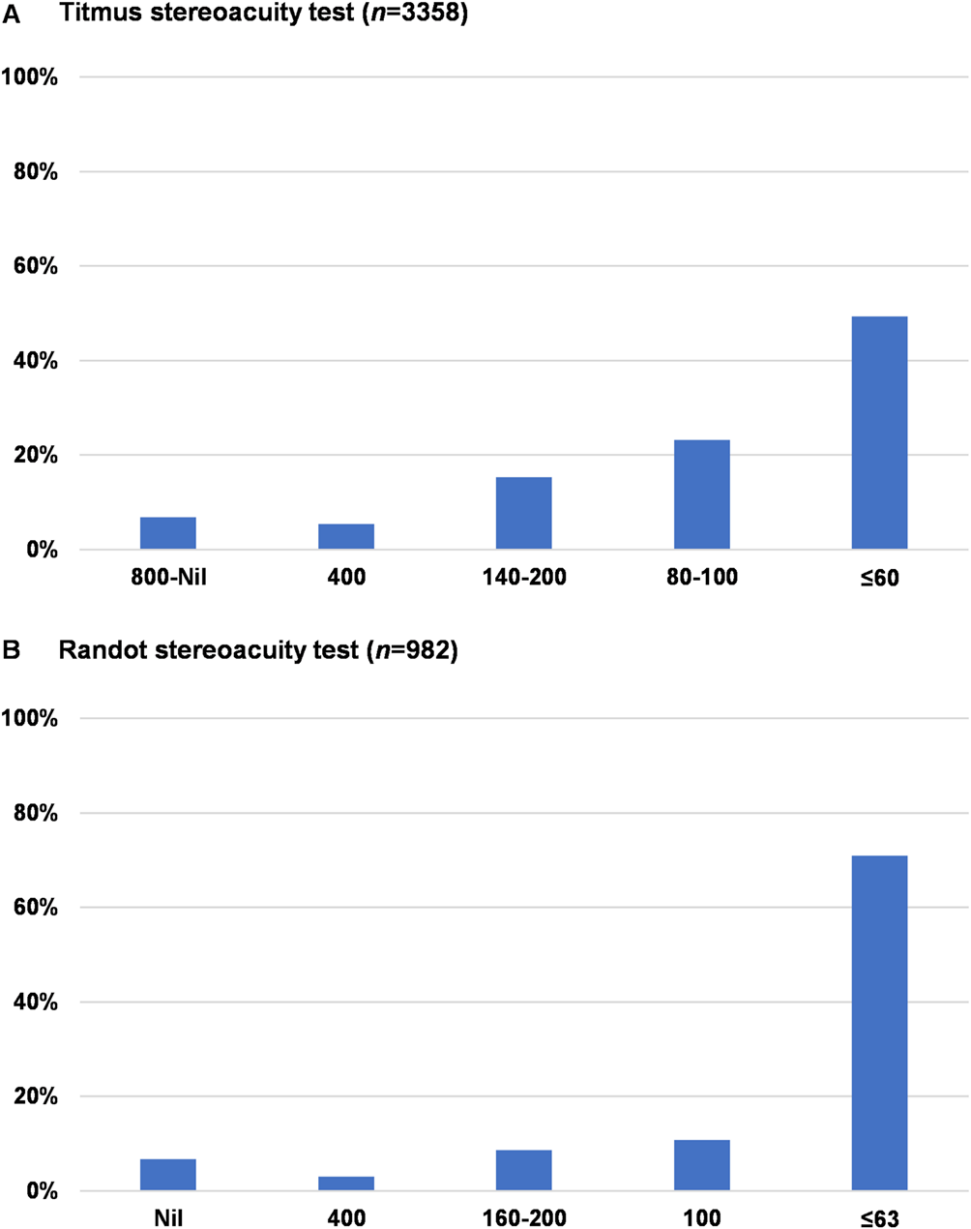
Histograms of near stereoacuity results. Most participants showed good stereoacuity results: 49.3% with ≤ 60.

Histograms of the Titmus (circles) and Randot stereo test results at 40 cm are shown in Fig. 1A and B. “Good stereopsis,” defined as ≤ 60 arcsec in the Titmus stereotest and as ≤ 63 arcsec in the Randot stereo test, was observed in 49.3% (1657/3358) and 71.0% (697/982), respectively.

## Discussion

This study described the objective examination findings from the KIEMS, which is one of the largest clinical studies on intermittent exotropia to date. Although many previous studies on the clinical characteristics of intermittent exotropia have been conducted, the KIEMS is expected to provide the most comprehensive and reliable overview of the clinical spectra of intermittent exotropia in terms of sample size and study parameters.

In this study, the number of female participants (51.9%) was comparable to that of male participants (48.1%). In a previous population-based cohort study including participants aged < 19 years in the United States, a female predominance (64.1%) was reported^19^. Another multicenter cohort study in the United Kingdom also reported a slight female predominance (55.9%) in children aged < 12 years with untreated intermittent exotropia^20^. In contrast, in Singaporean^4^ and Chinese^5^ population-based studies in children aged < 6 years (mostly of Chinese ethnicity), the prevalence of exotropia showed no sex difference when compared with the general population. In addition, a previous population-based study in Korea reported that sex was not significantly associated with clinically significant intermittent exotropia (≥ 15 PD) in adolescence^7^. Studies in Asian countries including our study have found no sex predominance in the prevalence of intermittent exotropia, whereas Western studies tended to show a female predominance. Future studies with age or ethnicity standardization are needed to clarify the sex differences in intermittent exotropia.

In this study, basic-type exotropia (86.2%) was the predominant type followed by convergence insufficiency-type (9.4%) and divergence excess-type (4.4%) exotropia when classified based on a ≥ 10 PD difference between the distant and near exotropia angles. Patients with the convergence insufficiency type were older than those with the other two types. Similarly, a recent study in Korea reported that basic-type exotropia was the most prevalent type (79.2%) in 355 patients with exotropia^14^. A population-based study from China reported a 74.7% prevalence of basic-type exotropia in 166 patients with intermittent exotropia aged 3–6 years^5^. Rutstein and Corliss also reported basic-type exotropia as the most common type in 73 patients^21^. A study from Singapore reported that divergence excess-type exotropia had a higher prevalence (59.5%) than basic-type exotropia (27%) in 453 patients with intermittent exotropia; however, the authors speculated that some patients with basic-type exotropia may have been inadvertently classified to the divergence excess type, as the children were not routinely patched to eliminate tenacious proximal fusion^22^. However, Burian and Franceschetti observed basic-type exotropia in 33% and convergence insufficiency-type exotropia in 55% of 237 prospectively collected consecutive patients, although they used stricter standards in classifying cases as convergence insufficiency-type exotropia^23^. Kushner and Morton observed divergence excess-type exotropia in 48.5%, which was the most prevalent type, although it included 80 patients (39.6% of the total participants) with simulated divergence excess (within a distant–near angle difference of 10 PD after 1 h of monocular patching), and basic-type exotropia in 38.6% of 202 patients with intermittent exotropia^24^. They reported that convergence insufficiency-type exotropia was more common in older participants, consistent with the current study (Table 4). The proportion of intermittent exotropia types may be affected by the inclusion criteria used or the clinical characteristics of the participants.

**Table 4:** Comparison among studies on intermittent exotropia types.

	Year	*n*	Age criteria	Age (mean [SD], years)	Basic		Convergence insufficiency		Divergence excess	
					Proportion	Age (years)	Proportion	Age (years)	Proportion	Age (years)
Current study	2021	5331	None	8.2 [7.6]	86.2%	8.0 [7.5]	9.4%	10.8 [8.1]	4.4%	7.0 [6.3]
Burian and Franceschetti^20^	1970	237	None	N/A	33%	12.6 (3–59)	55%	18.4 (5–61)	12%	9.5 (4–16)
Kushner and Morton^21^	1998	202	None	4–64 (mostly < 20)	38.6%	N/A	12.8%	N/A	48.5%	N/A
Rutstein and Corliss^18^	2003	73	None	20 (mean), 11 (median)	71.2%	N/A	13.6%	N/A	15.1%	N/A
Chia et al.^19^	2007	493	< 16 years	5.2 (median)	27%	5.6 [3.0]	10%	5.4 [3.0]	55%	6.8 [2.8]
Pan et al.^5^	2016	166	3–6 years	4.95 [0.72]	74.7%	N/A	5.4%	N/A	19.9%	N/A
Bae et al.^17^	2019	355	None	N/A	79.2%	5.5 [3.3]	3.7%	N/A	17.2%	5.1–5.2

*SD* standard deviation, *N/A* not applicable.

Alternating ocular dominance (48.3% at distance, 61.0% at near) was more common than right or left dominance (29.1% for the right eye and 22.6% for the left eye at distance; 22.1% for the right eye and 16.9% for the left eye at near) in this study. The proportion of alternating ocular dominance at near fixation was larger than that at distance fixation. Similarly, fusion control was better under the near viewing condition than under the distant viewing condition in this study. Previous studies investigating fusion control in patients with intermittent exotropia showed similar results^25–27^. In monocular dominance, there is a preference for one eye over the other eye under the binocular viewing condition, whereas no such preference exists in alternating ocular dominance^28^. It is well known that patients with intermittent exotropia rarely manifest amblyopia in either eye (if amblyopia occurs, it mostly manifests in the non-dominant eye) because the eyes can remain aligned at least in the near fixation condition^29^. Therefore, the result of this study confirmed that patients with intermittent exotropia show good binocular interaction.

More than 60% of the participants saw four or five lights in the distant Worth four-dot test, which suggests that patients with intermittent exotropia have relatively good binocular function at distant fixation, in which the sensory function of one eye does not overwhelm that of the other eye; however, seeing four lights in the test does not necessarily mean that the participants had central foveal fusion^30^. Monocular suppression was observed in < 40% of the patients, evenly in each eye. In the Titmus stereotest at 40 cm, approximately 50% of the participants showed good stereopsis of ≤ 60 arcsec, reflecting central fusion at near fixation. Moreover, in the Randot stereoacuity test at near fixation, > 70% of the participants showed ≤ 63 arcsec of stereopsis. Romanchuk et al. reported that 72.5% of their 109 patients showed better stereopsis than 60 arcsec in the Titmus stereo test even after ≥ 9 years follow-up from the initial visit^31^. Similarly, Mohney et al. reported that 63% of 152 patients showed 60 arcsec or better stereopsis in the Randot stereo test in a Pediatric Eye Disease Investigator Group study^32^. It is well known that patients with intermittent exotropia have relatively good near stereopsis^1^. The participants in this study can be assumed to have similarly good binocular functions, as previously reported.

This study should be viewed in the light of its limitations. Owing to the retrospective study design, data collection could not be performed as strictly as in a prospective study, which may have inevitably biased the patient selection or data collection process. Moreover, data were collected from 65 strabismus specialists from 53 different institutions and the circumstances of ophthalmologic examinations may have been different among the investigators, possibly affecting the study results. Despite efforts to reduce variability through the use of a standardized protocol and standardized case report forms, this study had the same limitations as many other multicenter studies.

In conclusion, this large observational study that included 5385 participants reported the objective findings of intermittent exotropia. In most of the study participants, intermittent exotropia was diagnosed during childhood (age, 8.2 ± 7.6 years). Basic-type exotropia was the most common type, followed by the convergence insufficiency and divergence excess types. In the assessment of fusion control, good to fair control was observed in 69.2% at distance fixation and in 79.8% at near fixation, and “good stereopsis” ($\le$ 60 arcsec in the Titmus stereotest and ≤ 63 arcsec in the Randot stereo test) was observed in 49.3% and 71.0%, respectively. This study potentially provides the most reliable information on the general clinical spectra of intermittent exotropia thus far, given the large study size and the coordination among many specialized investigators. Future studies using the KIEMS data are expected to provide more information about various aspects of intermittent exotropia.

## Methods

The KIEMS is a nationwide, retrospective, observational, cross-sectional, and multicenter study. The protocol of the KIEMS has been described elsewhere^18^. Briefly, the study was conducted as a collaboration among 65 strabismus specialists who were members of KAPOS and affiliated with 53 institutions in Korea. The medical records of patients who visited the eye clinic of each institution for the first time between March 1, 2019, and February 29, 2020, were reviewed. Participants with intermittent exotropia with ≥ 8 prism diopters (PD) at distance fixation (at 6 m) or near fixation (33 cm) in the prism and alternate cover test (PACT), regardless of age, were included in this study. Participants who had previous strabismus surgery history were excluded. Participants were excluded if they had signs of incomitant strabismus, ocular conditions affecting vision or prior ocular surgical history, chromosomal anomalies, or systemic disorders such as congenital anomalies or neurologic disorders. The KIEMS protocol conformed to the tenets of the Declaration of Helsinki. The protocol was approved by the Institutional Review Board of Kim’s Eye Hospital (KEH 2020-05-007) and by each participating institution. The requirement for informed consent was waived by the Institutional Review Board of Kim’s Eye Hospital because the study used retrospectively collected clinical data and the data were accessed anonymously.

The KIEMS collected data from subjective questionnaires completed by patients or guardians and from the results of objective ophthalmologic examinations conducted by strabismus specialists. In this study, we collected and analyzed the following objective data from ophthalmologic examinations in the KIEMS: age, sex, best-corrected visual acuity, refractive errors measured using cycloplegic refraction with 1% cyclopentolate hydrochloride (Cyclogyl; Alcon Lab. Inc., Fort Worth, TX, USA) and 1% tropicamide (Mydriacyl, Alcon Lab. Inc.), angles of deviation in PACT (in the primary, secondary, and head-tilted positions under distant [6 m] and near [33 cm] viewing conditions using accommodative targets with the patients’ best optical correction), and associated strabismus (e.g., dissociated vertical deviation, vertical deviation, and oblique muscle dysfunction). Vertical deviation was defined as hypertropia/hypotropia of ≥ 5 PD in the primary position. Lateral incomitance was defined as a decrease in the exo-angle of ≥ 20% in the right or left gaze, as compared with that in the primary position. “A” pattern exotropia was defined as a condition in which the exotropia angle at down gaze was higher by ≥ 10 PD than that at up gaze. Likewise, “V” pattern exotropia was defined as a condition in which the exotropia angle at up gaze was higher by ≥ 15 PD than that at down gaze. Right or left ocular dominance was determined to be present when the right or left eye had a shorter duration of dissociation during the uncover test, and alternating ocular dominance was identified when the duration of dissociation was similar between the two eyes. Fusion control under the distant and near viewing conditions was also investigated and classified as follows: good control, when ocular fusion was disrupted only after the cover test at distance fixation and was rapidly regained without blinking or fixating ocular movements; fair control, when ocular fusion was regained only after blinking or fixating movements after disruption with cover testing at distance fixation; and poor control, when ocular fusion was spontaneously broken without fusion disruption or was not regained despite blinking or refixation^33^. For sensory status evaluation, the Worth four-dot test (Richmond Products, Albuquerque, NM, USA) under the distant viewing condition and either the Titmus stereotest (Stereo Optical Co., Inc., Chicago, IL, USA) or Randot stereotest (Vision Assessment Corporation, Elk Grove Village, IL, USA) under the near viewing condition were performed. Stereoacuity of ≤ 60 arcsec in the Titmus stereotest or ≤ 63 arcsec in the Randot stereo test was defined as “good stereopsis.” More detailed findings of the ophthalmologic examinations are provided in an article describing the KIEMS methodology^18^.

Statistical analysis was performed using SPSS (version 21.0; IBM Corporation, Armonk, NY, USA). Statistical significance was set at *P* < 0.05. Bonferroni correction was applied to the *P* value for subgroup analyses. Mean ages were compared between male and female participants using an independent t-test. Exodeviation angles in the secondary positions and in the right and left head-tilted positions, compared with the exodeviation angle in the primary position, were analyzed using a paired t-test. The differences in the ratios of ocular dominance and fusion control at distant and near fixation conditions were compared using Pearson’s chi-square test.

## Data Availability

Data supporting the findings of the current study are available from the corresponding author upon reasonable request.
