# Author Correction: A cross-sectional study of ophthalmologic examination findings in 5385 Koreans presenting with intermittent exotropia

**DOI:** 10.1038/s41598-023-29515-x

**Published:** 2023-02-14

**Authors:** Dae Hee Kim, Jae Ho Jung, Mi Young Choi, Jeong‑Min Hwang, Su Jin Kim, Yeon‑hee Lee, Sueng‑Han Han, Dong Gyu Choi, The Korean Association of Pediatric Ophthalmology and Strabismus (KAPOS), Seung-Hee Baek, Hee-young Choi, Dong Cheol Lee, Se-Youp Lee, Han Woong Lim, Hyun Taek Lim, Key Hwan Lim, Won Yeol Ryu, Hee Kyung Yang, Hee-young Choi, Hyun Taek Lim, Seung-Hee Baek, Shin Hae Park, Haeng-Jin Lee, Sook-Young Kim, Se-Youp Lee, Hyo Jung Gye, So Young Kim, Sun Young Shin, Jihyun Park, Won Yeol Ryu, Hye Sung Park, Hae Jung Paik, Joo Yeon Lee, Hee Kyung Yang, Shin Yeop Oh, Soo Jung Lee, Seung Ah Chung, Jin Choi, Sei Yeul Oh, Mirae Kim, Young-Woo Suh, Nam Yeo Kang, Hae Ri Yum, Sun A. Kim, Hyuna Kim, Jinu Han, Yoonae A. Cho, Hyunkyung Kim, Helen Lew, Dong Cheol Lee, Sang Hoon Rah, Yung-Ju Yoo, Key Hwan Lim, Hyosook Ahn, Ungsoo S. Kim, Jung Ho Lee, Hokyung Choung, Seong-Joon Kim, Hyeshin Jeon, Hyun Jin Shin, So Young Han, Hwan Heo, Soochul Park, Songhee Park, Sung Eun Kyung, Changzoo Kim, Kyung-Ah Park, Eun Hye Jung, Eun Hee Hong, Han Woong Lim, Daye Choi, Youn Joo Choi, Nam Ju Moon, In Jeong Lyu, Soon Young Cho

**Affiliations:** 1 Department of Ophthalmology, Kim’s Eye Hospital, Seoul, Korea; 2 Department of Ophthalmology, Seoul National University College of Medicine, Seoul National University Hospital, Seoul, Korea; 3 Department of Ophthalmology, Chungbuk National University College of Medicine, Chungbuk National University Hospital, Cheongju, Korea; 4 Department of Ophthalmology, Seoul National University College of Medicine, Seoul National University Bundang Hospital, Seongnam, Korea; 5 Department of Ophthalmology, Pusan National University School of Medicine, Yangsan Pusan National University Hospital, Yangsan, Korea; 6 Department of Ophthalmology, Chungnam National University College of Medicine, Chungnam National University Hospital, Daejeon, Korea; 7 Department of Ophthalmology, Yonsei University College of Medicine, Severance Eye Hospital, Seoul, Korea; 8 Department of Ophthalmology, Hallym University College of Medicine, Kangnam Sacred Heart Hospital, 1, Singil-Ro, Yeongdeungpo-Gu, Seoul, 07441 Republic of Korea; 9 Department of Ophthalmology, Pusan National University Hospital, Busan, Korea; 10 Department of Ophthalmology, Asan Medical Center, Seoul, Korea; 11 Department of Ophthalmology, Seoul St. Mary’s Hospital, Seoul, Korea; 12 Department of Ophthalmology, Jeonbuk National University Hospital, Jeonju, Korea; 13 Department of Ophthalmology, Daegu Catholic University Medical Center, Daegu, Korea; 14 Department of Ophthalmology, Keimyung University DongSan Hospital, Daegu, Korea; 15 Department of Ophthalmology, Nune Eye Hospital, Seoul, Korea; 16 Department of Ophthalmology, Soonchunhyang University Cheonan Hospital, Cheonan, Korea; 17 Department of Ophthalmology, Nune Eye Hospital, Daegu, Korea; 18 Department of Ophthalmology, Dong-A University Hospital, Busan, Korea; 19 Department of Ophthalmology, Siloam Eye Hospital, Seoul, Korea; 20 Department of Ophthalmology, Gachon University Gil Medical Center, Incheon, Korea; 21 Department of Ophthalmology, Hallym University Sacred Heart Hospital, Anyang, Korea; 22 Department of Ophthalmology, Samsung Changwon Hospital, Changwon, Korea; 23 Department of Ophthalmology, Inje University Haeundae Paik Hospital, Busan, Korea; 24 Department of Ophthalmology, Ajou University Hospital, Suwon, Korea; 25 Department of Ophthalmology, Inje University Sanggye Paik Hospital, Seoul, Korea; 26 Department of Ophthalmology, Samsung Medical Center, Seoul, Korea; 27 Department of Ophthalmology, Korea University Ansan Hospital, Ansan, Korea; 28 Department of Ophthalmology, Bucheon St. Mary’s Hospital, Bucheon, Korea; 29 Department of Ophthalmology, Eunpyeong St. Mary’s Hospital, Seoul, Korea; 30 Department of Ophthalmology, Sungmo Eye Hospital, Busan, Korea; 31 Department of Ophthalmology, Soonchunhyang University Seoul Hospital, Seoul, Korea; 32 Department of Ophthalmology, Gangnam Severance Hospital, Seoul, Korea; 33 Department of Ophthalmology, Hangil Eye Hospital, Incheon, Korea; 34 Department of Ophthalmology, Bundang CHA Medical Center, Seongnam, Korea; 35 Department of Ophthalmology, Wonju Severance Christian Hospital, Wonju, Korea; 36 Department of Ophthalmology, Kangwon National University Hospital, Chuncheon, Korea; 37 Department of Ophthalmology, Ewha Womans University Mokdong Hospital, Seoul, Korea; 38 Daegu Premier Eye Center, Daegu, Korea; 39 Department of Ophthalmology, SMG-SNU Boramae Medical Center, Seoul, Korea; 40 Department of Ophthalmology, Konkuk University Medical Center, Seoul, Korea; 41 Department of Ophthalmology, Kangbuk Samsung Hospital, Seoul, Korea; 42 Department of Ophthalmology, Chonnam National University Hospital, Gwangju, Korea; 43 Department of Ophthalmology, Saevit Eye Hospital, Goyang, Korea; 44 Magok Dream Light Eye Clinic, Seoul, Korea; 45 Department of Ophthalmology, Kosin University Gospel Hospital, Busan, Korea; 46 Department of Ophthalmology, Nowon Eulji Medical Center, Seoul, Korea; 47 Department of Ophthalmology, Hanyang University Guri Hospital, Guri, Korea; 48 Department of Ophthalmology, Hanyang University Hospital, Seoul, Korea; 49 Department of Ophthalmology, Hallym University Kangdong Sacred Heart Hospital, Seoul, Korea; 50 Department of Ophthalmology, Chungang University Hospital, Seoul, Korea; 51 Department of Ophthalmology, Korea Cancer Center, Goyang, Korea; 52 Department of Ophthalmology, Dongguk University Hospital, Gyeongju, Korea

Correction to: *Scientific Reports* 10.1038/s41598-023-28015-2, published online 24 January 2023

The original version of the Article contained errors in the Consortia author list of The Korean Association of Pediatric Ophthalmology and Strabismus (KAPOS), where author names Dong Gyu Choi and Dae Hee Kim were duplicated in the KIEMS writing committee, and Jae Ho Jung, Mi Young Choi, Jeong-Min Hwang, Su Jin Kim, Yeon-hee Lee, Sueng-Han Han and Dong Gyu Choi were duplicated in KIEMS investigators in the KAPOS.

The original Article has been corrected.

